# Urinary and salivary endocrine measurements to complement Tanner staging in studies of pubertal development

**DOI:** 10.1371/journal.pone.0251598

**Published:** 2021-05-13

**Authors:** Mandy Goldberg, Anna J. Ciesielski Jones, John A. McGrath, Christie Barker-Cummings, Deborah S. Cousins, Lauren M. Kipling, Juliana W. Meadows, James S. Kesner, Michele Marcus, Carolyn Monteilh, Dale P. Sandler

**Affiliations:** 1 Epidemiology Branch, National Institute of Environmental Health Sciences, Research Triangle Park, North Carolina, United States of America; 2 Social & Scientific Systems, Inc., Durham, North Carolina, United States of America; 3 Department of Epidemiology, Rollins School of Public Health, Emory University, Atlanta, Georgia, United States of America; 4 Division of Applied Research and Technology, National Institute for Occupational Safety & Health, Cincinnati, Ohio, United States of America; 5 Departments of Environmental Health and Pediatrics, Emory University Schools of Public Health and Medicine, Atlanta, Georgia, United States of America; National Institute of Child Health and Human Development (NICHD), NIH, UNITED STATES

## Abstract

**Background:**

Many studies investigating pubertal development use Tanner staging to assess maturation. Endocrine markers in urine and saliva may provide an objective, sensitive, and non-invasive method for assessing development.

**Objective:**

Our objective was to examine whether changes in endocrine levels can indicate the onset of pubertal development prior to changes in self-rated Tanner stage.

**Methods:**

Thirty-five girls and 42 boys aged 7 to 15 years were enrolled in the Growth and Puberty (GAP) study, a longitudinal pilot study conducted from 2007–2009 involving children of women enrolled in the Agricultural Health Study (AHS) in Iowa. We collected saliva and urine samples and assessed pubertal development by self-rated Tanner staging (pubic hair, breast development (girls), genital development (boys)) at three visits over six months. We measured dehydroepiandrosterone (DHEA) in saliva and creatinine-adjusted luteinizing hormone (LH), testosterone, follicle stimulating hormone (FSH), estrone 3-glucuronide (E_1_3G) and pregnanediol 3-glucuronide (Pd3G) concentrations in first morning urine. We evaluated the relationships over time between Tanner stage and each biomarker using repeated measures analysis.

**Results:**

Among girls still reporting Tanner breast stage 1 at the final visit, FSH levels increased over the 6-month follow-up period and were no longer lower than higher stage girls at the end of follow-up. We observed a similar pattern for testosterone in boys. By visit 3, boys still reporting Tanner genital stage 1 or pubic hair stage 1 had attained DHEA levels that were comparable to those among boys reporting Tanner stages 2 or 3.

**Conclusions:**

Increasing concentrations of FSH in girls and DHEA and testosterone in boys over a 6-month period revealed the start of the pubertal process prior to changes in self-rated Tanner stage. Repeated, non-invasive endocrine measures may complement the more subjective assessment of physical markers in studies determining pubertal onset.

## Introduction

Ages at onset of pubertal milestones have decreased over time, with a particularly steep recent decline in age at breast development in girls [1, 2]. Earlier puberty is associated with anxiety, depression and earlier onset of risk behaviors in adolescence [3, 4] and increased risks of chronic diseases, including testicular cancer in men and breast cancer in women [5–7]. Given the potential burden of early puberty on a population level, epidemiologic studies assessing pubertal onset are needed to identify risk factors for early puberty and its short- and long-term health consequences.

Puberty is a process that occurs over 3–4 years on average and includes hormonal changes, increases in somatic growth, the development of secondary sexual characteristics and attainment of reproductive maturity [8]. Pubertal development in humans is driven by two independent processes, adrenarche and gonadarche [9]. Adrenarche, the activation of the hypothalamic-pituitary-adrenal (HPA) axis, typically occurs around 6–8 years of age, leading to increased production of adrenal androgens and the development of axillary and pubic hair [10]. Gonadarche, the re-activation of the hypothalamic-pituitary-gonadal (HPG) axis, often occurs after adrenarche and triggers the production of sex steroids [8, 9]. In boys, increasing levels of testosterone stimulate genital development [11]. In girls, increasing estrogens, including estradiol and estrone, stimulate breast development and menarche while progesterone is essential for attaining reproductive capacity and also influences breast maturation [10–12]. The normal age range for pubertal onset is 8–13 years in girls and 9–14 years in boys [13].

Most epidemiologic studies rely on age at menarche or assessment of physical markers to determine pubertal development, commonly Tanner staging. Tanner staging of pubic hair development, breast development (girls), and genital development (boys) is based on visual assessment using a scale from 1 (no development) to 5 (adult maturity), with Tanner stage (TS) 2 indicating the onset of development [14, 15]. Palpation is recommended to increase the accuracy of breast staging, particularly in overweight girls, and quantitative measurement of testicular volume with an orchidometer can improve genital staging in boys [16]. While Tanner staging by trained clinical raters is often considered the gold standard in epidemiologic studies, the collection of repeated clinical assessments is resource-intensive and infeasible for many studies. Concerns and embarrassment around physical exams may hinder study participation [17]. Parental or self-assessments based on TS drawings and descriptions are less invasive and easier to administer, but less accurate compared with clinical assessments [17–19].

Increases in circulating hormone levels precede and drive the appearance of physical markers of development [20, 21]. Overnight peaks of blood serum gonadotropins and sex steroid hormones are sensitive in detecting pubertal onset in girls [22] and boys [23], but repeated overnight serum measurements are infeasible outside a clinical setting. The invasiveness and inconvenience of collecting serum make it of limited use for longitudinal population-based studies. Hormones or their metabolites measured in urine and saliva samples, which are less invasive to collect, may be more acceptable to participants and are highly correlated with serum measures [24–26]. Urine measures are less subject to variability due to the pulsatile secretion of hormones that may affect single serum measurements, minimizing misclassification and increasing the statistical power of the urinary measurement [24, 26, 27].

Previous studies have shown that hormones are correlated with age and TS [24–26, 28–34], but many have been cross-sectional [25, 26, 28, 29, 32]. We conducted a longitudinal pilot study to evaluate the feasibility and utility of using repeated assessments of objective endocrine biomarkers to complement more subjective self-reports of pubertal development in population-based research. Our aims were to examine repeated measures of salivary and urinary endocrine markers by sex, TS and age, and to assess whether changes in biomarkers over six months reveal pubertal onset prior to changes in self-reported TS. We also examined whether changes in endocrine markers predict progression to a higher TS during follow-up.

## Materials and methods

### Study design and population

The Growth and Puberty (GAP) study was a longitudinal pilot study involving children of women enrolled in the Agricultural Health Study (AHS) in Iowa [35]. The AHS is a prospective study of licensed pesticide applicators (mostly male) and their spouses (mostly female) from Iowa and North Carolina. Mothers enumerated children in their household who were under age 21 at enrollment and reported on subsequent births in follow-up interviews. Questionnaires are available at www.aghealth.nih.gov.

Families were eligible for inclusion if they resided in a county within 100 miles of and including Des Moines (excluding Cedar Rapids and Iowa City), the mother completed the AHS Female and Family Health Questionnaire or the Female Follow-up questionnaire, and at least one child was 7–15 years of age as of January 1, 2007. At that time, potentially eligible children lived at least 3 months per year at the AHS address; were able to stand unassisted; were the biological, adoptive or step child of the AHS participant, and lived with their biological mother at least part of the year. Potential participants were randomly selected from eligible families. Once a child in the target age range was selected, all eligible siblings were also invited to participate. Eligible families were mailed a study brochure and later contacted by study staff.

Of the 213 families mailed a brochure, 79 (37%) were successfully contacted for screening. Six families were found ineligible, 19 refused, and 54 were enrolled. Nine families withdrew prior to the first visit due to child refusal (6) or other reasons (3). Seventy-seven children from the remaining 45 families completed the study.

Visits were conducted from May 2007-April 2009. Families were visited three times over six months at 3-month intervals. At each visit, a parental questionnaire and child interview were administered, a morning saliva sample and evening and first morning urine samples were retrieved, self- and parent-assessed TS forms were collected, and height and weight were measured. Parents gave written consent and children gave verbal assent to participate. The Institutional Review Board of the National Institute of Environmental Health Sciences, the University of Iowa Institutional Review Board, and the Copernicus Group Institutional Review Board approved the study.

### Hormone assays

Salivary dehydroepiandrosterone (DHEA) and urinary luteinizing hormone (LH), testosterone, and creatinine were measured in boys and salivary DHEA and urinary follicle stimulating hormone (FSH), estrone 3-glucuronide (E_1_3G), pregnanediol 3-glucuronide (Pd3G), and creatinine in girls. Details of the specimen collection and assays are in S1 Appendix.

### Assessment of pubertal development

At visit 1 (V1), children were given TS drawings with age-appropriate descriptions enclosed in an envelope and asked to use a mirror for self-examination in a private room and mark the drawing most closely matching their development. Forms were returned to the envelope by the child and sealed. For subsequent visits, children were asked to complete TS forms ahead of time without parental assistance and place them in a sealed envelope for the interviewer. Parents of children ages 7–10 years were asked to complete TS forms as best they could without examining their child. Parental assessment of older children was deemed unacceptable by the Institutional Review Board. We calculated percent agreement between parental and self-reports for 30 parent-child pairs with available data. We used child’s self-reported data for the statistical analyses since this data was collected for all participants.

### Statistical analysis

We examined the distribution of age by TS and calculated mean endocrine levels by TS and age. We combined the 5 stages into 3 groups due to small numbers in some stages: TS-1 (pre-pubertal), TS-2/3 (early pubertal) and TS-4/5 (late pubertal). For example, 3 girls reported breast stage (BS)-2 and no girls reported BS-5 at V3. These categories have been used in prior research [31].

We used the TS at V3 as the outcome for each child to compare changes in biomarker levels over six months among children that were pre-pubertal, early pubertal or late pubertal at V3. We used the first morning urine measure to reflect overnight increases in hormone secretion and allow for comparison with previous studies [24–26]. Measurements below the lower limit of detection (LOD) were replaced with LOD/√2.

We used linear regression to examine if a change in endocrine levels preceded Tanner-based puberty onset, defined as TS≥2. For children with final (V3) TS of 1, we used repeated measures analysis to regress log-transformed endocrine levels on visit number to determine whether endocrine markers increased prior to a progression in TS. We also regressed endocrine markers separately at each visit on final TS group to determine whether children remaining at TS-1 at V3 had lower endocrine levels than children reporting higher stages. We calculated body mass index (BMI) based on measured weight and height at the initial visit as the weight in kilograms divided by the height in meters squared. We then calculated age- and sex-specific BMI percentiles and Z-scores based on the 2000 United States Centers for Disease Control and Prevention (CDC) growth charts to account for differences in BMI by age and sex [36]. We adjusted all models for BMI Z-score at V1 and accounted for within-family correlation using generalized estimating equations. We tested for statistical significance of differences in endocrine markers by visit and TS group using the Wald test from the repeated measures analyses adjusted for BMI Z-score.

We used generalized linear mixed models to examine whether change in endocrine levels from V1 to V3 predicted progressing at least one TS over the same time period, adjusting for BMI z-score and TS at the first visit (pre-pubertal vs pubertal). We used logistic regression without accounting for clustering when mixed models showed evidence of insufficient familial variance. We conducted analyses using SAS 9.3 [37].

We excluded 2 participants with missing TS at V3. All children provided urinary and salivary samples at each visit. However, 8 saliva samples (5 at V1, 1 at V2 and 2 at V3) could not be tested due to insufficient quantity or had no DHEA detected in the sample and were excluded from DHEA analyses.

We conducted sensitivity analyses examining all five stages and using the maximum endocrine marker level (evening or morning) instead of the first morning void for urinary measures. In addition, we repeated the analyses examining FSH and E_1_3G in relation to breast development excluding girls with a BMI ≥85^th^ percentile to consider the likelihood that misclassification of breast development in overweight girls explained the observed differences in endocrine markers since it can be difficult to distinguish glandular from fat tissue in overweight girls [16].

## Results

The mean ages of the 42 boys and 35 girls enrolled in the study were 11.9 and 11.7 years, respectively (Table 1). Parents were white, except for two fathers who were multiracial.

**Table 1:** Characteristics of study population at initial visit by gender.

	Boys (n = 42)		Girls (n = 35)	
	Mean	Range	Mean	Range
Age (Years)	11.9	7.6–15.5	11.7	7.2–14.7
Pre-menarchal at Visit 1	-	-	10.3	7.2–14.0
Post-menarchal at Visit 1	-	-	13.7	12.1–14.7
Height (cm)	152.2	123.0–186.0	150.1	122.0–170.0
Height-for-Age Percentile^a^	57.5	1.2–98.9	59.2	4.6–97.7
Weight (kg)	48.1	23.3–95.6	44.5	22.6–73.7
Weight-for-Age Percentile^a^	66.8	7.3–99.6	59.6	0.8–97.3
Body Mass Index (kg/m^2^)	20.3	14.7–30.2	19.3	13.5–27.0
Body Mass Index-for-age Percentile^a^,^b^	67.4	2.2–98.8	55.3	3.0–98.6

^a^Age- and sex-specific percentiles calculated based on the 2000 United States Centers for Disease Control and Prevention growth charts

^b^29% of boys and 26% of girls had a BMI-for-age percentile ≥85.

The mean ages of children rating themselves as pre-pubertal at V3 was less than 10 years for each pubertal marker (Table 2). Mean age increased with TS, though age ranges overlapped for adjacent categories.

**Table 2:** Mean age by child self-reported Tanner stage at third (final) visit.

Tanner Stage	Mean age in years; Range (N)	
	Boys	Girls
Pubic Hair		
1	9.9; 8.2–11.5 (8)	9.6; 7.6–11.1 (10)
2&3	11.9; 9.0–13.7 (16)	11.7; 10.2–14.5 (8)
4&5	14.3; 12.7–15.8 (16)	14.0; 12.0–15.2 (17)	
Genitals (Boys) / Breasts (Girls)		
1	9.9; 8.2–11.5 (7)	9.8; 7.6–11.0 (11)
2&3	12.0; 9.0–14.1 (19)	12.5; 8.6–14.6 (10)
4&5	14.3; 12.1–15.8 (14)	13.9; 10.3–15.2 (14)

Across visits, percent agreement between parental and self-reports ranged from 79–100% for breast development and 86–100% for pubic hair development in girls, and 60–71% for genital development and 57–87% for pubic hair development in boys.

Tanner self-ratings were positively correlated with all endocrine levels at V3 (Table 3). This pattern was also observed at earlier visits (S1 and S2 Tables). Endocrine levels increased with age.

**Table 3:** Mean endocrine marker concentrations at visit 3 by Tanner stage.

Tanner Stage^b^	Mean (CI) (N)								
	Boys				Girls				
		Saliva	Urine^a^			Saliva	Urine^a^		
	N	DHEA (pg/ml)	LH (mIU/mg Cr)	Testosterone (ng/mg Cr)	N	DHEA (pg/ml)	FSH (mIU/mg Cr)	Estrone (E_1_3G) (ng/mg Cr)	Pregnanediol (Pd3G) (μg/mg Cr)
Pubic Hair									
1	8	121.8 (-75.3,319.0) (8)	1.1 (0.3,2.0) (8)	6.0 (2.3,9.8) (8)	10	51.2 (21.2,81.2) (10)	3.3 (2.1,4.5) (10)	3.4 (1.5,5.3) (10)	1.4 (1.1,1.7) (10)
2&3	16	119.4 (55.5,183.2) (16)	2.3 (1.3,3.4) (16)	11.4 (5.5,17.4) (16)	8	93.3 (53.9,132.6) (7)	4.6 (0.7,8.5) (8)	12.8 (-0.8,26.4) (8)	3.0 (0.3,5.8) (8)
4&5	16	173.1 (109.2,236.9) (16)	3.8 (3.0,4.6) (16)	32.3 (22.5,42.1) (16)	17	151.2 (113.0,189.4) (16)	3.6 (2.5,4.8) (17)	15.9 (9.1,22.7) (17)	3.6 (1.9,5.3) (17)
Genitals (Boys) / Breasts (Girls)									
1	7	136.3 (-95.7,368.3) (7)	1.2 (0.1,2.2) (7)	6.7 (2.5,10.8) (7)	11	57.5 (26.3,88.7) (11)	3.8 (2.6,5.0) (11)	3.5 (1.8,5.2) (11)	1.4 (1.1,1.6) (11)
2&3	19	131.0 (67.1,194.9) (19)	2.6 (1.6,3.6) (19)	12.5 (6.8,18.2) (19)	10	87.8 (59.7,115.8) (8)	3.9 (0.9,6.9) (10)	17.0 (6.1,27.9) (10)	4.8 (1.9,7.6) (10)
4&5	14	157.5 (95.7,219.2) (14)	3.5 (2.7,4.2) (14)	33.2 (22.1,44.3) (14)	14	160.7 (119.0,202.3) (14)	3.6 (2.3,5.0) (14)	14.2 (6.2,22.1) (14)	2.6 (1.3,3.9) (14)

^a^Endocrine markers assessed in first morning urine samples.

^b^Self-report of Tanner stage at visit 3.

**Fig 1:**
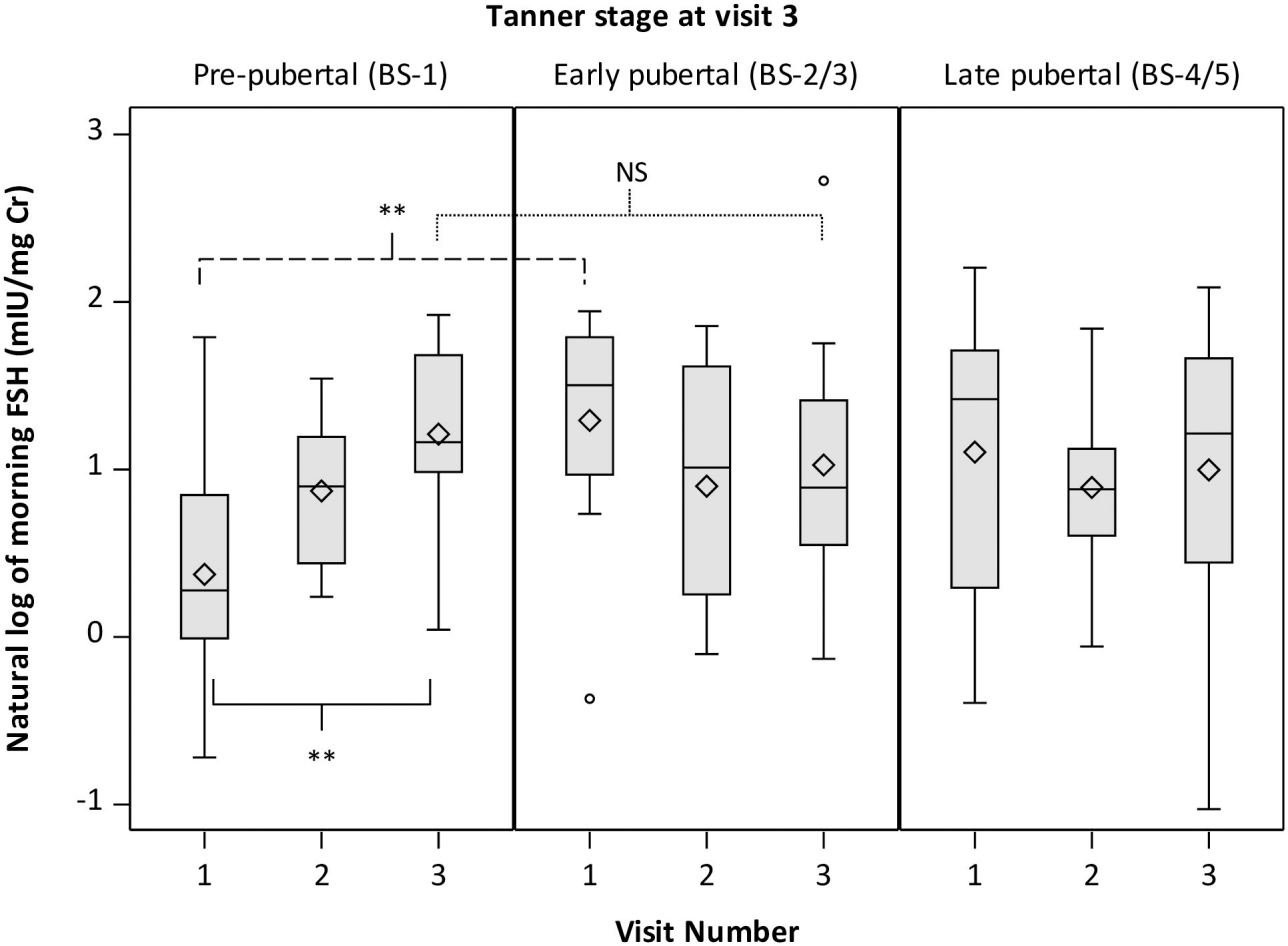
Morning urinary FSH levels in girls by Tanner breast stage and visit. Girls are classified as pre-pubertal (BS-1), early pubertal (BS-2/3) or late pubertal (BS-4/5) based on self-report of Tanner breast stage at visit 3. P values from repeated measures analysis adjusted for BMI Z-score shown for V3 vs. V1 levels among BS-1 girls (solid), BS-2/3 vs. BS-1 at V1 (dashed) and BS-2/3 vs. BS-1 at V3 (dotted). NS = p≥0.05; *p<0.05; **p<0.01.

Urinary FSH increased over time in girls prior to self-reports of breast development (Fig 1). For girls still reporting BS-1 at V3, FSH levels were significantly higher at V3 than at V1 (p<0.0001). At V1, FSH levels were significantly lower for girls that remained pre-pubertal than for girls reaching early pubertal (p<0.0001 for BS-2/3 vs. BS-1) or late pubertal stages by V3 (p<0.01 for BS-4/5 vs. BS-1). At V3, girls still rating themselves as BS-1 had FSH levels not significantly different from those in children in higher stages (p = 0.51 for BS-2/3 vs. BS-1 and p = 0.47 for BS-4/5 vs. BS-1). These patterns were less pronounced for pubic hair development (S1 Fig).

Girls still reporting BS-1 at V3 had higher levels of E_1_3G at V3 compared with V1, but their levels of E_1_3G at V3 were still significantly lower than the levels of early pubertal and late pubertal girls (S2 Fig). This pattern was also observed for pubic hair development (S1 Fig). Pd3G did not increase over time in girls that remained BS-1 or pubic hair stage (HS)-1 by V3 (S1 and S2 Figs). Pd3G was higher at V3 for girls in BS-2/3 versus BS-1 girls. There were no differences in DHEA levels over time in pre-pubertal girls or between pre-pubertal and early-pubertal girls at V1 or V3 (S1 and S2 Figs).

Compared with girls who were pre-menarcheal at V3 (n = 17), girls who experienced menarche prior to V1 (n = 14) had higher TS ratings (p <0.0001) and higher concentrations of E_1_3G and Pd3G (p<0.05) at each visit, and higher FSH at V1 only (p<0.05).

**Fig 2:**
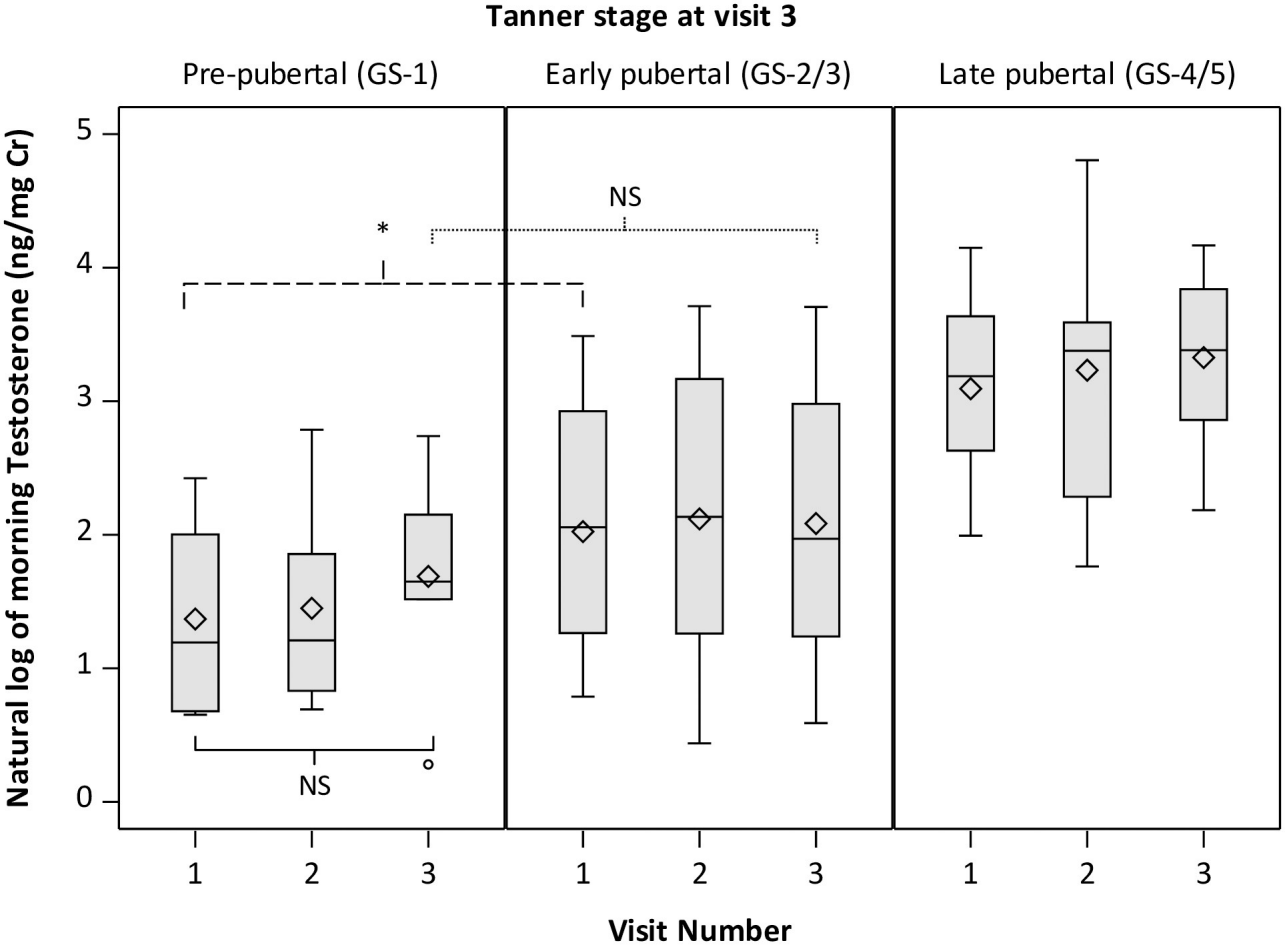
Morning urinary testosterone levels in boys by Tanner genital stage and visit. Boys are classified as pre-pubertal (GS-1), early pubertal (GS-2/3) or late pubertal (GS-4/5) based on self-report of Tanner genital stage at visit 3. P values from repeated measures analysis adjusted for BMI Z-score shown for V3 vs. V1 levels among GS-1 boys (solid), GS-2/3 vs. GS-1 at V1 (dashed) and GS-2/3 vs. GS-1 at V3 (dotted). NS = p≥0.05; *p<0.05; **p<0.01.

**Fig 3:**
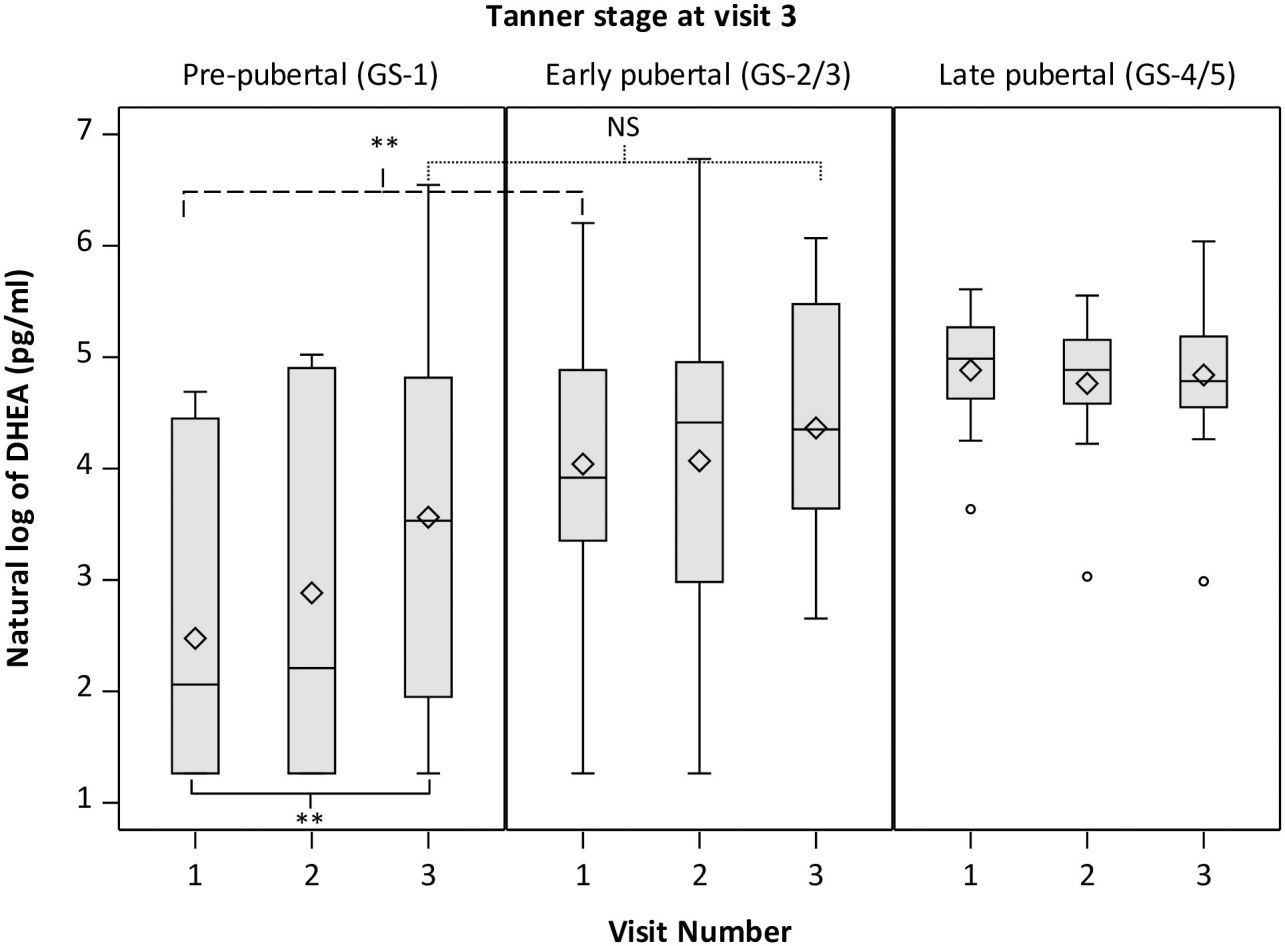
Salivary DHEA levels in boys by Tanner genital stage and visit. Boys are classified as pre-pubertal (GS-1), early pubertal (GS-2/3) or late pubertal (GS-4/5) based on self-report of Tanner genital stage at visit 3. P values from repeated measures analysis adjusted for BMI Z-score shown for V3 vs. V1 levels among GS-1 boys (solid), GS-2/3 vs. GS-1 at V1 (dashed) and GS-2/3 vs. GS-1 at V3 (dotted). NS = p≥0.05; *p<0.05; **p<0.01.

Testosterone levels in boys that remained at GS-1 at V3 increased over time (p = 0.05) (Fig 2). Testosterone levels in pre-pubertal boys were lower at V1 but did not significantly differ at V3 compared with those in early pubertal boys, though levels were still lower than late pubertal boys at V3 (p<0.0001). DHEA levels also rose significantly over visits for boys who remained at GS-1 at V3 (p<0.001 for V1 vs V3). At V3 DHEA levels for GS-1 boys no longer significantly differed from GS-2/3 boys (p = 0.20) but were lower than DHEA levels for boys at GS-4/5 (p = 0.04) (Fig 3). LH did not increase over time in pre-pubertal boys and was lower at V1 and V3 compared with boys in later stages of genital development (S2 Fig). Similar patterns were observed for pubic hair development (S1 Fig).

Patterns were similar in sensitivity analyses using all five stages or the maximum of evening and first morning urine measures. The associations between FSH and E_1_3G and breast TS were similar in analyses restricted to non-overweight girls.

Changes in endocrine levels between V1 and V3 were not associated with increases in genital TS, breast TS, or pubic hair TS over follow-up (S3 Table).

## Discussion

Consistent with prior work [24–26, 28–34], we observed that endocrine markers increased with age and TS. We also found that endocrine markers increased prior to self-reports of pubertal development based on physical markers. Only FSH in girls and testosterone and DHEA in boys increased in children still rating themselves as TS-1 at the end of the study and no longer differed from the more stable levels of early pubertal children. Rising levels of these objective biomarkers suggest the onset of the pubertal process prior to self-reports of physical development and may also help to distinguish between children rating themselves as pre-pubertal or in early puberty.

Adrenarche in both sexes stimulates the production of DHEA, which plays a role in pubic hair development [9]. DHEA did not increase from V1 to V3 in pre-pubertal girls and levels did not differ between pre-pubertal and early pubertal girls throughout follow-up, suggesting that short-term changes in DHEA would not be a useful biomarker of pubertal onset in girls. However, we detected increases in salivary DHEA prior to the onset of genital and pubic hair development in boys. Long-term increases in adrenal androgens may be more useful than short-term measures in predicting pubertal onset in girls. For example, Biro *et al* found that serum measures of DHEA-S first increased between 30 and 18 months prior to the onset of breast development [38]. DHEA exhibits diurnal patterns and is less stable than DHEA-S [39], which also may explain why we did not observe any consistent patterns in DHEA in girls.

FSH and LH are produced by the pituitary gland and stimulate the gonads to produce sex steroid hormones [10]. Previous cross-sectional analyses have observed increases in urinary FSH and/or LH in boys and girls with age and TS [25, 26, 32], but few studies have examined longitudinal changes in gonadotropins. We found that FSH increased in girls over the 6-month follow-up period prior to self-reports of breast development, while we did not observe increases in LH in pre-pubertal boys. Singh *et al* observed increases in urinary LH in 10–12 year old boys and girls followed for one-year with LH assessed every 3 months and positive correlations of LH with TS across the follow-up period, but did not determine if changes in LH over time differed by pubertal status [24]. Kolby *et al* found that urinary LH, but not FSH, could distinguish between girls with central precocious puberty (CPP) and girls with premature breast development without activation of the HPG axis, and that urinary LH decreased over 12 weeks in girls with CPP after treatment with a gonadotropin-releasing hormone agonist [26]. This study suggested that urinary LH could be useful in diagnosing and managing girls with disordered puberty [26], but did not assess changes in LH or FSH in children with normal puberty.

Estrogen in girls and testosterone in boys are produced by the gonads and stimulate breast and genital development, respectively [10]. We found that testosterone increased in boys prior to self-reports of genital development with comparable levels in pre- and early-pubertal boys after six months of follow-up. Estrone levels in pre-pubertal girls, despite increasing over follow-up, remained lower than those in girls with reported breast development. Increases in serum estrone were first observed between 12–18 months prior to the onset of breast development in a longitudinal study of peri-pubertal girls in Cincinnati, with estrone levels continuing to increase up to a year after breast development [30, 38]. This study also evaluated longitudinal changes in serum estradiol and testosterone in girls, both of which first increased 6–12 months before breast development, about 6 months later than estrone [38]. Two longitudinal studies of Australian boys and girls observed positive correlations of urinary and/or serum measures of estradiol and testosterone with TS across one-year [24] and three-year follow-up periods [31], respectively. Few children in these studies, who were age 9 or older at baseline, still rated themselves as TS1 at the end of follow-up, and they did not examine patterns of change in sex steroids specifically among pre-pubertal children [24, 31].

Within-individual increases in endocrine markers over six months were not associated with progression to a higher TS in girls or boys. Our analysis of pubertal progression was likely underpowered to detect an association between increases in endocrine markers over time and advancements in TS. Most participants remained in the same TS over the six-month follow-up, suggesting that a longer time frame may be necessary to identify predictors of pubertal progression. In an Australian study of 10–12 year-olds followed for one year, changes in urinary LH, estradiol and testosterone were not associated with changes in TS, though one-year changes in serum estradiol and testosterone levels in males were associated with pubertal progression [24].

All participants completed all three visits, supporting the feasibility of collecting repeated biospecimens and pubertal assessments in children ages 7–15 years. The assessment of DHEA (an adrenal androgen), gonadotropins and sex steroids allowed us to compare endocrine patterns over time and by TS. Although our sample size was small, statistical power was higher in repeated measures analyses since we incorporated three data points per individual.

Although compliance was excellent, overall participation was low. This may reflect perceived sensitivity of the nature of the questions and examinations. The study protocol originally included a clinical TS assessment, but this was eliminated due to feedback from families who refused participation, including concerns about exam invasiveness and inconvenience of a clinic visit. Low participation rates could reflect that farm families may have limited free time and considered the requirement of repeated in-person visits overly burdensome, particularly for older children. Findings may not be generalizable to non-agricultural populations since pesticide exposure in this cohort may affect hormone levels and pubertal timing [40] or to other racial/ethnic groups, as almost all participants were white.

We used LOD/√2 for measures with levels below the LOD which could have affected comparisons between groups with differing frequencies of missing values. Pre-pubertal children were more likely to have endocrine measures <LOD than early or late pubertal children. For boys in GS-1, the percent of samples <LOD at each visit ranged from 14–30% for DHEA and 40–57% for LH and testosterone. For girls in BS-1, all samples were ≥LOD for DHEA, FSH and Pd3G, but 42–60% of samples at each visit were <LOD for E_1_3G. Despite the relatively high proportion of samples <LOD for LH, testosterone and E_1_3G, we still observed a trend of increasing levels in pre-pubertal children across the visits, except for LH in boys.

We could not evaluate the validity of self-reported TS compared to clinical staging since we did not obtain physical examinations performed by trained practitioners. Although the validity of self-reported TS has varied across studies (for reviews, see [17, 19]), the accuracy of self-assessments is higher in distinguishing between pre-pubertal (TS-1) and pubertal (TS≥2) status [29, 41]. We collapsed the five stages into three groups, which may improve accuracy. Studies comparing endocrine measures with clinician, self and parent TS assessments found that biomarkers correlated with TS across all three sources of assessment [29, 33, 34]. Our results were similar for breast/genital and pubic hair staging, even though breast and genital development result from gonadarche and pubic hair development is initiated by adrenarche and augmented by gonadarche [9]. While this could reflect a tendency of adolescents to report the same TS for both markers, breast/genital and pubic hair development sometimes occur concurrently [42].

Sample collection was not timed to the menstrual cycle in post-menarchal girls. Sampling during different phases of the cycle could cause large fluctuations in endocrine markers, particularly Pd3G. Since pre-pubertal and most early pubertal girls had yet to experience menarche, comparisons between these groups are likely not affected by timing of sample selection. Girls do not typically establish regular ovulatory cycles until at least 1–2 years after menarche [43]. The median time since menarche at V3 in our sample was 1.7 years (range 0.3–4.1). We did not measure LH in girls and could not examine changes in LH or the LH:FSH ratio in relation to TS.

## Conclusions

Urinary FSH in girls and salivary DHEA and urinary testosterone in boys increased prior to self-reports of pubertal development. Repeated measures of these endocrine markers over six months identified patterns that distinguished pre-pubertal from early pubertal children based on TS ratings and were more informative than measures at single points in time. Collecting longitudinal urine and saliva samples is non-invasive and can be done outside of a clinic setting. Identifying risk factors for early puberty is a research priority, but large-scale epidemiologic studies often rely on self-assessments of pubertal development, which are prone to error and may bias estimates of associations between exposures and pubertal onset. Our results suggest that epidemiologic studies should collect repeated endocrine measures, in addition to more subjective measures of pubertal development like Tanner staging, to help identify pubertal onset, particularly in settings where it is not feasible to conduct clinical exams. Replication of our findings in larger, diverse studies and studies with clinical staging of pubertal development is warranted to support the use of short-term changes in endocrine markers as biomarkers of pubertal onset in population-based research.

## Supporting information

S1 Appendix Supplemental methods. Additional details regarding specimen collection, storage and analyses.
(PDF)

S1 Table Mean endocrine marker concentrations at visit 1 by Tanner stage. (PDF)

S2 Table Mean endocrine marker concentrations at visit 2 by Tanner stage. (PDF)

S3 Table Associations between change in endocrine biomarker levels from V1 to V3 and pubertal progression (increase of ≥1 Tanner stage over the follow-up period). (PDF)

S1 Fig Distributions of log-transformed endocrine marker levels by visit and Tanner pubic hair stage. Children are classified as pre-pubertal (HS-1), early pubertal (HS-2/3) or late pubertal (HS-4/5) based on self-report of Tanner pubic hair stage at visit 3. P values from repeated measures analysis adjusted for BMI Z-score shown for V3 vs. V1 levels among HS-1 (solid), HS-2/3 vs. HS-1 at V1 (dashed) and HS-2/3 vs. HS-1 at V3 (dotted). NS = p≥0.05; *p<0.05; **p<0.01.
(PDF)

S2 Fig Distributions of log-transformed endocrine marker levels by visit and Tanner stage (genital stage for boys and breast stage for girls). Children are classified as pre-pubertal (BS/GS-1), early pubertal (BS/GS-2/3) or late pubertal (BS/GS-4/5) based on self-report of Tanner breast stage (girls) or genital stage (boys) at visit 3. P values from repeated measures analysis adjusted for BMI Z-score shown for V3 vs. V1 levels among BS/GS-1 (solid), BS/GS-2/3 vs. BS/GS-1 at V1 (dashed) and BS/GS-2/3 vs. BS/GS-1 at V3 (dotted). NS = p≥0.05; *p<0.05; **p<0.01.
(PDF)

## Abbreviations

AHS: Agricultural Health Study
BMI: body mass index
BS[1–5]: Tanner Breast Stages 1 thru 5
CPP: central precocious puberty
DHEA: dehydroepiandrosterone
E_1_3G: estrone 3-glucuronide
FSH: follicle stimulating hormone
GAP: Growth and Puberty study
GS[1–5]: Tanner Genital Stages 1 thru 5
HPA: hypothalamic-pituitary-adrenal
HPG: hypothalamic-pituitary-gonadal
HS[1–5]: Tanner Hair Stages 1 thru 5
LH: luteinizing hormone
LOD: limit of detection
Pd3G: pregnanediol 3-glucuronide
TS: Tanner stage
V[1–3]: Visits 1–3
