# Supplementary material for: Urinary and salivary endocrine measurements to complement Tanner staging in studies of pubertal development
Source: PLoS One. 2021 May 13;16(5):e0251598. doi: 10.1371/journal.pone.0251598 (PMC8118248; doi:10.1371/journal.pone.0251598)
Supplement: S1 Appendix — Additional details regarding specimen collection, storage and analyses. (PDF) [file pone.0251598.s001.pdf]

S1 Appendix. Supplemental Methods.

Specimen Collection: Saliva. To obtain peak DHEA levels, participants were instructed to collect morning saliva samples. On the morning of each study visit, children were to expectorate 2 mL of saliva through a short plastic straw into a Nunc Cryovial. Collection instructions were based on the Passive Drool Method designed by Salimetrics, LLC (State College, PA). During saliva collection, children were asked to imagine that they were eating, smelling, and chewing their favorite food and to move their jaws as if they were eating. Children were asked to collect the saliva sample prior to brushing their teeth or consuming food or drink. However, if these conditions could not be met, children were asked to rinse their mouths with water and wait 10 minutes before collecting saliva. If the child had a body temperature over 102° F or bleeding oral lesions, the parents were asked to reschedule the study visit.

Specimen Collection: Urine. Participants were instructed to collect their urine samples between 6 and 9 pm on the evening prior to the visit and during their first void on the morning of the visit. If either of these collections were missed, the child was instructed to collect a sample as close to the preferred time as possible. Urine samples were collected in LeakBuster Specimen Containers (Starplex Scientific Inc., Etobicoke, Ontario). Parents were asked to document the date and time of collection and the time the child woke on that morning.

Specimen Storage and Transport. Parents were asked to refrigerate the saliva and urine samples until the interviewer arrived to collect them later that day. The interviewer then transported samples using a portable refrigerator (Koolatron; Brantford, Ontario) plugged into the car lighter and kept at approximately 22°C below ambient levels. Once at the interviewer's home, the urine samples were stored at 4°C in a refrigerator and the saliva samples were stored in the freezer portion of the refrigerator. Samples were shipped overnight in insulated containers to the Social & Scientific Systems, Inc. (SSS) laboratory in North Carolina. Urine samples were shipped on frozen gel packs (-20°C) and saliva samples were shipped on dry ice. The average time from sample voiding to reception at the SSS lab was 6 days (range 2-16).

Urine analyses. Upon receipt, 5-ml urine aliquots were created in cryovials and preserved with 7% glycerol to prevent freeze-induced activity loss of luteinizing hormone (LH) and follicle-stimulating hormone (FSH).[1] Aliquots were stored at -80°C. Prior to analyses, duplicates were made from 10% of the samples for quality control assessment and interspersed among the samples. One set of aliquots was sent by express courier on dry ice to the National Institute for Occupational Safety and Health (NIOSH) Reproductive Endocrinology Laboratory and stored at -80°C until assayed.

LH and FSH were assayed in duplicate using non-competitive two-site time-resolved immunofluorometric assays (Perkin-Elmer cat. no. A031-101 and A017-201), modified for urine analyses.[2, 3] Urinary estrone 3-glucuronide ($E_1$3G) and pregnanediol 3-glucuronide (Pd3G) were assayed in triplicate using competitive double-antibody time-resolved

fluoroimmunoassays.[4] Total testosterone concentrations were measured in duplicate in hydrolyzed urine samples using a solid-phase radioimmunoassay (TKTT; Siemens). To measure the low testosterone levels in many of these peripubertal samples, we added an 8 ng/dl calibration point. Urinary creatinine was measured from duplicate 1:30 dilutions using a Vitros 250 Chemistry Analyzer (Ortho-Clinical Diagnostics).[5, 6] Urinary endocrine concentrations were adjusted for creatinine concentrations to normalize for urine dilution.

Saliva analyses. Upon receipt in NC, saliva samples were stored at -80°C. At the end of each of the three data collection periods, samples were shipped to Salimetrics to be assayed for dehydroepiandrosterone (DHEA) concentrations. All saliva samples were thawed and then centrifuged at 3,000 rpm for 10 minutes to remove mucin. Samples were screened for blood contamination and pH was determined. Samples testing outside the pH range of 4-9 were diluted in phosphate-buffered saline to correct pH prior to testing for salivary cortisol. Samples were assayed in duplicate using a double-antibody radioimmunoassay developed at the Penn State Behavioral Endocrinology Laboratory.[7]

References

1. Kesner JS, Knecht EA, Krieg Jr EF. Stability of urinary female reproductive hormones stored under various conditions. Reprod Toxicol. 1995;9(3):239-44. doi: 10.1016/0890-6238(95)00005-u.
2. Kesner JS, Knecht EA, Krieg EF, Jr. Time-resolved immunofluorometric assays for urinary luteinizing hormone and follicle stimulating hormone. Anal Chim Acta. 1994;285:13-22.
3. Kesner JS, Knecht EA, Krieg EF, Jr., Wilcox AJ, O'Connor JF. Detecting pre-ovulatory luteinizing hormone surges in urine. Human Reproduction. 1998;13(1):15-21.
4. Kesner JS, Knetch EA, Krieg EF, Jr., Barnard G, Mikola H, Kohen F, et al. Validation of time-resolved fluoroimmunoassays for urinary estrone 3-glucuronide and pregnanediol 3-glucuronide. Steroids. 1994;58:205-11.
5. Findlay J, Wu A, Knott V, Mauck L, Frickey P, Norton G. Development of a Kodak Ektachem clinical chemistry slide for CK-B activity. Clin Chem. 1985;31(1000).
6. Mauck J, Mauck L, Novros J, Norton G, Toffaletti J. Development of a single slide Kodak Ektachem® thin-film assay for serum and urine creatinine. Clin Chem. 1986;32:1197-8.
7. Granger DA, Schwartz EB, Booth A, Curran M, Zakaria D. Assessing dehydroepiandrosterone in saliva: a simple radioimmunoassay for use in studies of children, adolescents and adults. Psychoneuroendocrinology. 1999;24(5):567-79.
