# Supplementary material for: Urinary and salivary endocrine measurements to complement Tanner staging in studies of pubertal development
Source: PLoS One. 2021 May 13;16(5):e0251598. doi: 10.1371/journal.pone.0251598 (PMC8118248; doi:10.1371/journal.pone.0251598)
Supplement: S1 Table — (PDF) [file pone.0251598.s002.pdf]

S1 Table. Mean endocrine marker concentrations at visit 1 by Tanner stage.

		Mean (95% CI) (N)							
		Boys				Girls			
		Saliva	Urine[a]			Saliva	Urine[a]		
Tanner Stage[b]	N	DHEA (pg/ml)	LH (mIU/mg Cr)	Testosterone (ng/mg Cr)	N	DHEA (pg/ml)	FSH (mIU/mg Cr)	Estrone ($E_1$3G) (ng/mg Cr)	Pregnanediol (Pd3G) (μg/mg Cr)
Pubic Hair									
1	17	51.3 (20.8,81.8) (16)	1.1 (0.6,1.6) (17)	7.0 (2.8,11.1) (17)	13	58.3 (27.0,89.6) (12)	2.2 (1.0,3.3) (13)	2.1 (1.2,3.0) (13)	1.3 (1.0,1.6) (13)
2&3	15	108.8 (65.0,152.6) (14)	2.6 (1.4,3.8) (14)	13.7 (8.8,18.7) (14)	8	85.3 (36.7,133.9) (8)	4.2 (2.3,6.0) (8)	5.7 (3.1,8.3) (8)	1.4 (1.0,1.7) (8)
4&5	9	197.5 (96.4,298.5) (9)	4.0 (2.7,5.2) (9)	32.1 (18.9,45.3) (9)	14	215.3 (58.2,372.5) (12)	4.2 (2.6,5.7) (14)	14.9 (8.1,21.8) (14)	2.1 (1.5,2.7) (14)
Genitals (Boys) / Breasts (Girls)									
1	10	41.7 (7.1,76.3) (10)	1.4 (0.7,2.1) (10)	4.9 (2.8,6.9) (10)	12	42.8 (22.9,62.6) (11)	1.9 (0.9,2.8) (12)	2.3 (1.3,3.2) (12)	1.4 (1.1,1.6) (12)
2&3	20	107.0 (48.5,165.5) (18)	2.4 (1.3,3.6) (19)	12.3 (7.7,16.8) (19)	15	182.1 (49.0,315.3) (14)	4.2 (3.2,5.2) (15)	8.9 (5.0,12.8) (15)	1.6 (1.2,2.0) (15)
4&5	11	161.7 (112.3,211.0) (11)	2.8 (2.0-3.6) (11)	29.0 (17.2,40.7) (11)	8	135.1 (38.0,232.1) (7)	4.3 (1.3,7.3) (8)	15.1 (2.6,27.6) (8)	2.2 (1.1,3.3) (8)

[a]Endocrine markers assessed in first morning urine samples

[b]Self-report of Tanner stage at visit 1
