# Supplementary material for: Urinary and salivary endocrine measurements to complement Tanner staging in studies of pubertal development
Source: PLoS One. 2021 May 13;16(5):e0251598. doi: 10.1371/journal.pone.0251598 (PMC8118248; doi:10.1371/journal.pone.0251598)
Supplement: S2 Table — (PDF) [file pone.0251598.s003.pdf]

S2 Table. Mean endocrine marker concentrations at visit 2 by Tanner stage.

		Mean (95% CI) (N)							
		Boys				Girls			
		Saliva	Urine[a]			Saliva	Urine[a]		
Tanner Stage[b]	N	DHEA (pg/ml)	LH (mIU/mg Cr)	Testosterone (ng/mg Cr)	N	DHEA (pg/ml)	FSH (mIU/mg Cr)	Estrone ($E_1$3G) (ng/mg Cr)	Pregnanediol (Pd3G) (µg/mg Cr)
Pubic Hair									
1	12	33.9 (0.8,67.1) (12)	1.1 (0.5,1.8) (12)	5.0 (2.0,7.9) (12)	11	34.2 (23.9,44.5) (10)	2.2 (1.7,2.7) (11)	2.1 (1.0,3.2) (11)	1.3 (1.1,1.6) (11)
2&3	20	110.6 (77.5,143.8) (20)	2.7 (1.9,3.4) (20)	14.4 (9.6,19.2) (20)	9	137.4 (-1.5,276.2) (9)	3.8 (2.4,5.1) (9)	10.7 (4.3,17.0) (9)	2.9 (-0.7,6.6) (9)
4&5	9	227.5 (36.2,418.7) (9)	4.8 (2.9,6.7) (9)	45.8 (20.6,71.0) (9)	15	135.7 (80.2,191.1) (15)	2.6 (1.8,3.4) (15)	16.0 (9.0,23.1) (15)	3.4 (1.8,5.0) (15)
Genitals (Boys) / Breasts (Girls)									
1	7	34.2 (-15.1,83.5) (7)	1.0 (0.4,1.6) (7)	5.0 (0.3,9.7) (7)	10	49.4 (17.8,81.1) (9)	2.6 (1.8,3.4) (10)	2.4 (1.1,3.6) (10)	1.3 (1.0,1.5) (10)
2&3	24	127.8 (53.6,202.0) (24)	2.5 (1.8,3.3) (24)	14.1 (9.1,19.0) (24)	15	109.4 (29.8,188.9) (15)	3.4 (2.5,4.3) (15)	10.8 (5.5,16.1) (15)	1.5 (1.3,1.7) (15)
4&5	9	130.0 (83.8,176.1) (9)	4.3 (2.0,6.5) (9)	36.7 (8.7,64.7) (9)	9	167.9 (80.7,255.0) (9)	2.1 (1.2,2.9) (9)	17.1 (6.3,27.9) (9)	4.5 (2.1,7.0) (9)

[a]Endocrine markers assessed in first morning urine samples
[b]Self-report of Tanner stage at visit 2
