# Supplementary material for: Urinary and salivary endocrine measurements to complement Tanner staging in studies of pubertal development
Source: PLoS One. 2021 May 13;16(5):e0251598. doi: 10.1371/journal.pone.0251598 (PMC8118248; doi:10.1371/journal.pone.0251598)
Supplement: S3 Table — (PDF) [file pone.0251598.s004.pdf]

S3 Table. Associations between change in endocrine biomarker levels from V1 to V3 and pubertal progression (increase of ≥1 Tanner stage over the follow-up period).

	**Boys**	
Endocrine biomarker[a]	Pubic Hair OR (95% CI)[b]	Genitals OR (95% CI)[b]
Salivary DHEA (pg/ml)	1.00 (0.99, 1.01)[c]	1.00 (1.00, 1.01)[c]
Urinary LH (mIU/mg Cr)	1.09 (0.57, 2.08)[c]	0.83 (0.42, 1.62)[c]
Urinary Testosterone (ng/mg Cr)	1.04 (0.92, 1.18)[c]	1.07 (0.95, 1.20)[c]
	**Girls**	
	Pubic Hair OR (95% CI)[b]	Breasts OR (95% CI)[b]
Salivary DHEA (pg/ml)	1.00 (1.00, 1.01)[c]	0.97 (0.95, 1.00)
Urinary FSH (mIU/mg Cr)	1.10 (0.85, 1.43)[c]	1.05 (0.81, 1.35)
Urinary Estrone ($E_1$3G) (ng/mg Cr)	1.03 (0.96, 1.11)[c]	1.03 (0.96, 1.11)
Urinary Pregnanediol (Pd3G) (μg/mg Cr)	1.09 (0.82, 1.44)[c]	1.37 (0.94, 2.01)

[a]Urinary endocrine markers were assessed in first-morning urine samples.

[b]Odds ratio for a 1-unit change in the endocrine biomarker from V1 to V3. Models are adjusted for BMI Z-score and Tanner stage (pre-pubertal (TS1) vs. pubertal (TS≥2) at V1.

[c]Odds ratios from logistic regression model not accounting for familial variance. If not indicated, odds ratios from a generalized linear mixed model.
