# Supplementary material for: Urinary and salivary endocrine measurements to complement Tanner staging in studies of pubertal development
Source: PLoS One. 2021 May 13;16(5):e0251598. doi: 10.1371/journal.pone.0251598 (PMC8118248; doi:10.1371/journal.pone.0251598)
Supplement: S1 Fig — Children are classified as pre-pubertal (HS-1), early pubertal (HS-2/3) or late pubertal (HS-4/5) based on self-report of Tanner pubic hair stage at visit 3. P values from repeated measures analysis adjusted for BMI Z-score shown for V3 vs. V1 levels among HS-1 (solid), HS-2/3 vs. HS-1 at V1 (dashed) and HS-2/3 vs. HS-1 at V3 (dotted). NS = p≥0.05; *p<0.05; **p<0.01. (PDF) [file pone.0251598.s005.pdf]

S1 Fig. **Distributions of log-transformed endocrine marker levels by visit and Tanner pubic hair stage.** Children are classified as pre-pubertal (HS-1), early pubertal (HS-2/3) or late pubertal (HS-4/5) based on self-report of Tanner pubic hair stage at visit 3. P values from repeated measures analysis adjusted for BMI Z-score shown for V3 vs. V1 levels among HS-1 (solid), HS-2/3 vs. HS-1 at V1 (dashed) and HS-2/3 vs. HS-1 at V3 (dotted). NS = p≥0.05; *p<0.05; **p<0.01.

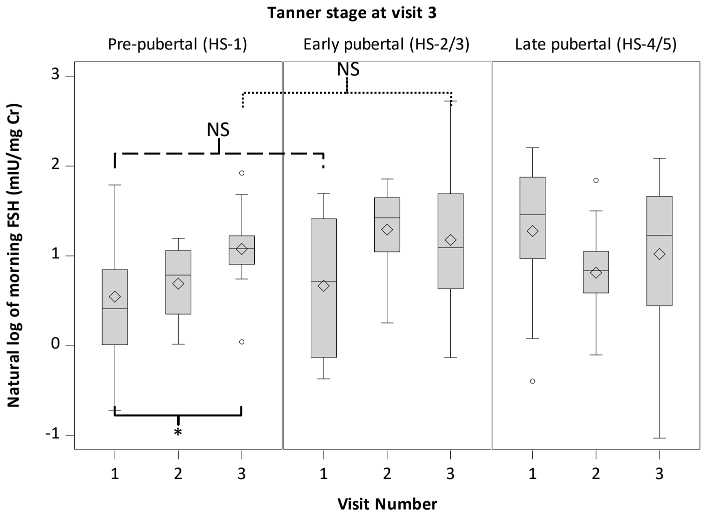

(a) Girls; urinary FSH

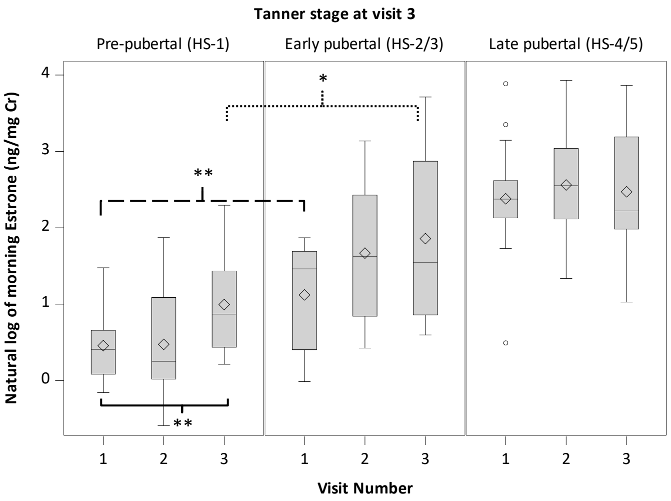

(b) Girls; urinary Estrone (E_13G)

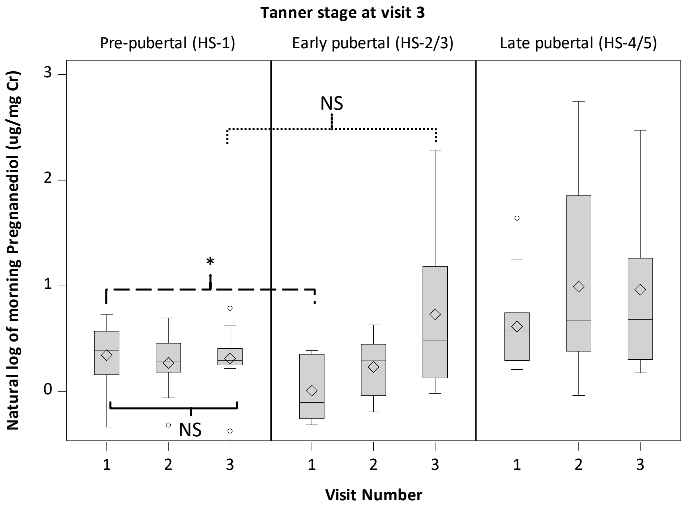

(c) Girls; urinary Pregnanediol (Pd3G)

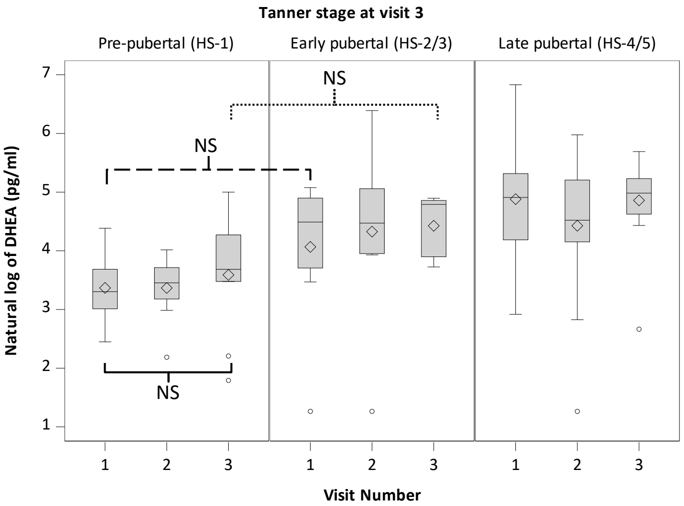

(d) Girls; salivary DHEA

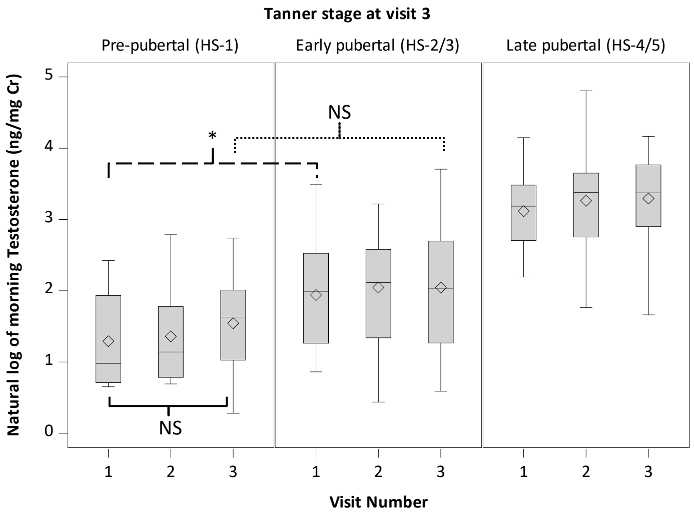

(e) Boys; urinary Testosterone.

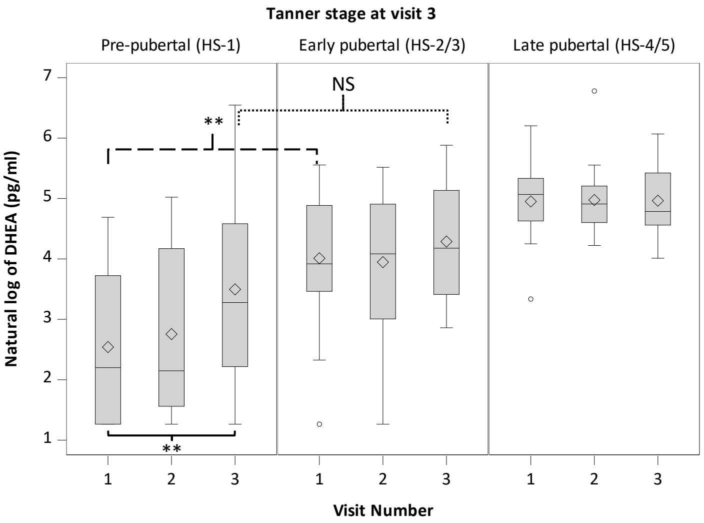

(f) Boys; salivary DHEA

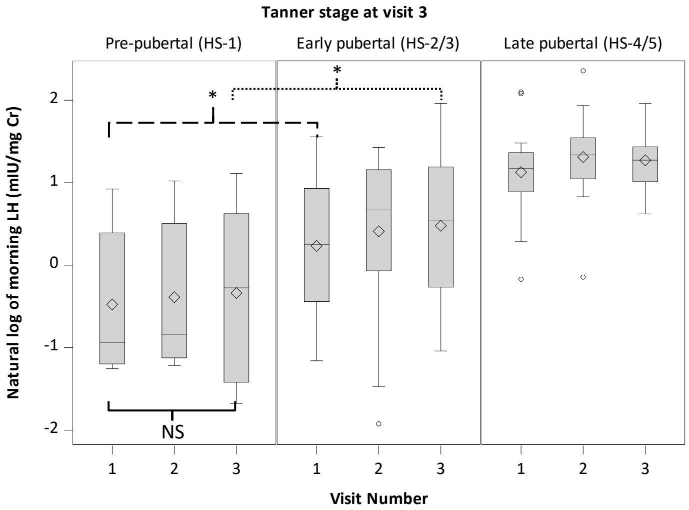

(g) Boys; urinary LH.
