# Supplementary material for: Urinary and salivary endocrine measurements to complement Tanner staging in studies of pubertal development
Source: PLoS One. 2021 May 13;16(5):e0251598. doi: 10.1371/journal.pone.0251598 (PMC8118248; doi:10.1371/journal.pone.0251598)
Supplement: S2 Fig — Children are classified as pre-pubertal (BS/GS-1), early pubertal (BS/GS-2/3) or late pubertal (BS/GS-4/5) based on self-report of Tanner breast stage (girls) or genital stage (boys) at visit 3. P values from repeated measures analysis adjusted for BMI Z-score shown for V3 vs. V1 levels among BS/GS-1 (solid), BS/GS-2/3 vs. BS/GS-1 at V1 (dashed) and BS/GS-2/3 vs. BS/GS-1 at V3 (dotted). NS = p≥0.05; *p<0.05; **p<0.01. (PDF) [file pone.0251598.s006.pdf]

S2 Fig. Distributions of log-transformed endocrine marker levels by visit and Tanner stage (genital stage for boys and breast stage for girls). Children are classified as pre-pubertal (BS/GS-1), early pubertal (BS/GS-2/3) or late pubertal (BS/GS-4/5) based on self-report of Tanner breast stage (girls) or genital stage (boys) at visit 3. P values from repeated measures analysis adjusted for BMI Z-score shown for V3 vs. V1 levels among BS/GS-1 (solid), BS/GS-2/3 vs. BS/GS-1 at V1 (dashed) and BS/GS-2/3 vs. BS/GS-1 at V3 (dotted). NS = p≥0.05; *p<0.05; **p<0.01.

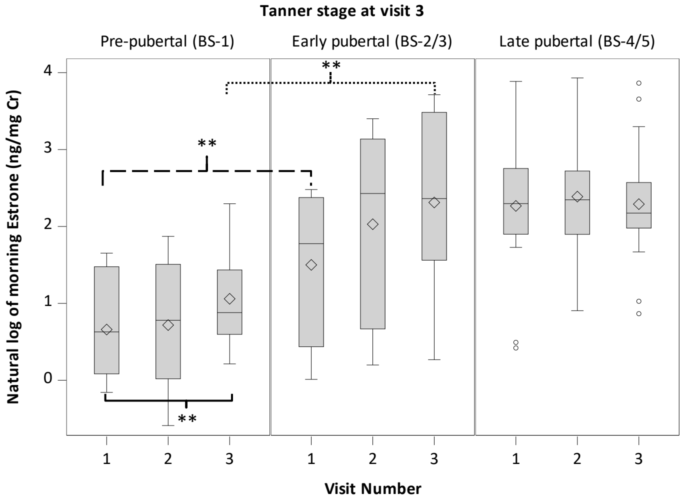

(a) Girls; urinary Estrone (E_13G)

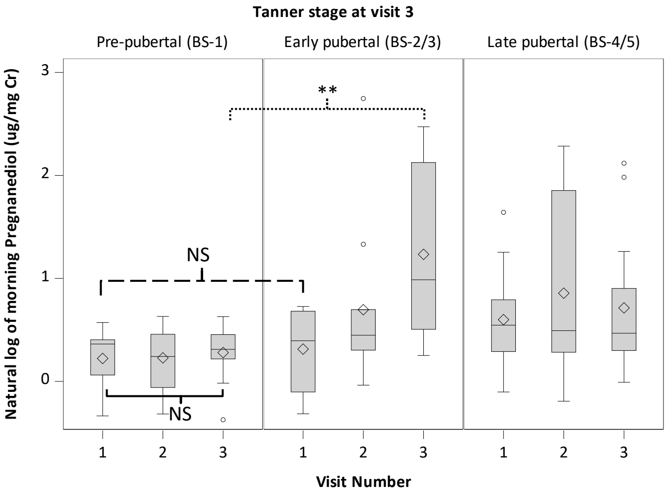

(b) Girls; urinary Pregnanediol (Pd3G)

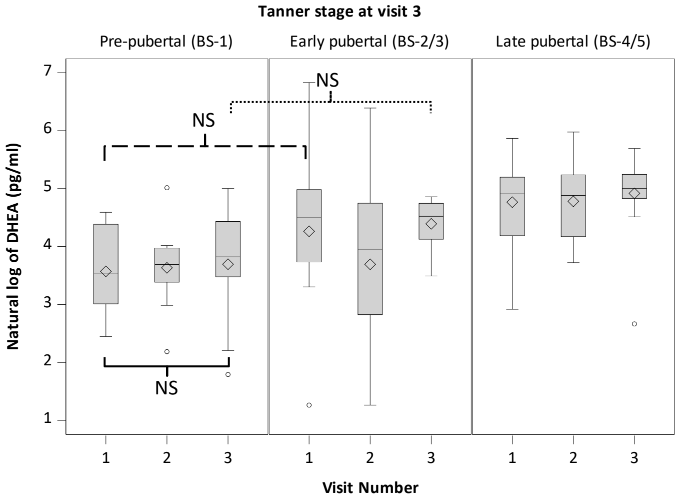

(c) Girls; salivary DHEA

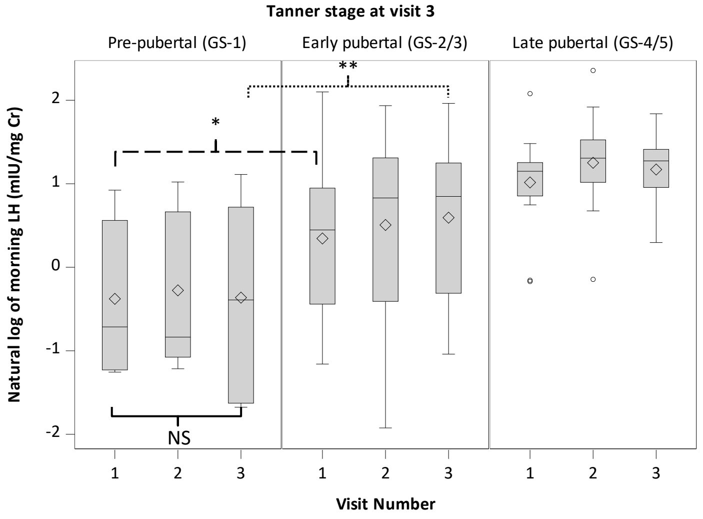

(d) Boys; urinary LH
